# Serum exosomal miR-4772-3p is a predictor of tumor recurrence in stage II and III colon cancer

**DOI:** 10.18632/oncotarget.12841

**Published:** 2016-10-24

**Authors:** Chang Liu, Cathy Eng, Jianjun Shen, Yue Lu, Yoko Takata, Amir Mehdizadeh, George J. Chang, Miguel A. Rodriguez-Bigas, Yanan Li, Ping Chang, Yixiang Mao, Manal M. Hassan, Fangyu Wang, Donghui Li

**Affiliations:** ^1^ Department of Gastrointestinal Medical Oncology, The University of Texas MD Anderson Cancer Center, Houston, Texas, USA; ^2^ Department of Surgical Oncology, The University of Texas MD Anderson Cancer Center, Houston, Texas, USA; ^3^ Department of Epigenetics and Molecular Carcinogenesis, The University of Texas MD Anderson Cancer Center, Smithville, Texas, USA; ^4^ Department of Gastroenterology and Hepatology, Jinling Hospital, Southern Medical University, Nanjing, China

**Keywords:** exosome, microRNA, colon cancer, qRT-PCR, Illumina RNA sequencing

## Abstract

**Purpose:**

The study was aimed to evaluate the prognostic or predictive value of serum exosomal microRNAs (miRNAs) for tumor recurrence and response to adjuvant therapy in stage II and stage III colon cancer.

**Results:**

145 differentially expressed mature miRNAs were identified (*P*<0.05) and 10 top hits were carried forward in validation test. MiR-4772-3p was significantly under-expressed in 27 patients with recurrence compared to in 57 patients without recurrence (*P*=0.002). The reduced expression was significantly related to increased risk of tumor recurrence and risk of death. As a predictor for tumor recurrence, ROC analysis revealed the AUC (95% CI) was 0.72 (0.59-0.85, *P*=0.001) for lower level of miR-4772-3p compared to 0.63 (0.51-0.75, *P*=0.062) for tumor site and 0.65 (0.51-0.78,*P*=0.034) for lymph node status. Among 66/84 patients who received FOLFOX adjuvant therapy, 9/10 (90%) patients with a lower level and 10/56 (18%) patients with a higher level of miR-4772-3p had tumor recurrence (*P*<0.001).

**Materials and Methods:**

Blood samples were prospectively collected from84 patients with stage II/III colon cancer after tumor resection and before adjuvant therapy. Serum exosomal miRNA profiles were determined by RNA sequencing. Differentially expressed mature miRNAs were identified between patients with or without tumor recurrence. The top hits were validated in individual RNA samples using quantitative real-time reverse transcription PCR.

**Conclusions:**

Reduced expression of serum exosomal miR-4772-3p is a prognostic biomarker for tumor recurrence in stage II and stage III colon cancer patients. The predictive value of this marker for response to FOLFOX adjuvant therapy needs further investigation.

## INTRODUCTION

Colorectal cancer (CRC) is the third most common cancer and the third leading cause of cancer death for men and women individually and the second leading cause for men and women combined in the USA [1]. Half of all CRC patients will present with stage II or stage III disease, and 20%-50% of them will develop disease recurrence. Of those, 74% will develop recurrence within the first 3 years of diagnosis, despite curative surgical intervention and adjuvant chemotherapy [2, 3]. Although clinic-pathological risk factors such as T4 or N2 disease, poorly differentiated histology, and suboptimal lymph node dissection are considered poor prognostic factors for recurrence, no predictive markers have been identified for patients who are likely to benefit from adjuvant chemotherapy [4–8]. Despite the FDA approval of Oncotype DX as a prognostic tool for locally advanced colon cancer, it is not heavily utilized as it has no predictive value for benefit of adjuvant chemotherapy. Biomarkers that could add both prognostic and predictive value to existing clinico-pathological risk factors is urgently needed for locally advanced disease, notably stage II colon cancer [9, 10].

MicroRNAs (miRNAs), the small non-coding RNAs that are associated with the development of cancer [11], have been shown to be potential biomarkers in various types of cancer, including CRC [12]. Examinations of miRNA profiles in tumor versus normal tissues and in the circulating blood have resulted in the discovery of various miRNAs that are associated with tumor progression and patient survival [9, 13–15]. Exosomes have been attracting major interest as potential diagnostic and prognostic biomarkers of cancer. Exosomes can be isolated from many body fluids, including both plasma and serum [16]. Tumor-derived exosomes are emerging as mediators of metastasis [17]. The isolation of cancer-specific exosomes in body fluids could enable the identification of DNA, RNA, miRNA, and proteins that aid in the treatment and management of cancer [18]. The role of serum exosomal miRNA as a prognostic marker for CRC recurrence was explored in one reported study conducted in six patients [19]. In this small study, one miRNA, miR-19a, was identified as a prognostic marker.

The purpose of this study was to prospectively evaluate the prognostic and predictive value of serum exosomal miRNA in patients with stage II and III colon cancer utilizing RNA sequencing of serum exosomal miRNAs, followed by a quantitative reverse transcription polymerase chain reaction (qRT-PCR) assay.

## RESULTS

Eighty-four patients were included in the study, with a median follow-up duration of 51 months (interquartile range: 45-64 months). Twenty-seven patients (32.1%) developed recurrent disease (14 local and 13 metastatic). The median age of the study population was 57 years. There was no significant difference in the distribution of demographics and major clinical factors between patients with or without tumor recurrence except number of positive lymph nodes and tumor location (Table 1). The recurrent group had a significantly higher frequency of >5 metastatic lymph nodes than did the non-recurrent group (*P*=0.03). The recurrent group also had a higher frequency of tumors of the sigmoid and ascending (right side) colon and a lower frequency of tumors of the cecum, transverse and the descending (left side) colon than did the non-recurrent group (*P*=0.029). Forty-seven out of 57 (82%) of the non-recurrent patients and 19/27 (70%) of the recurrent patients received FOLFOX adjuvant chemotherapy, 8/57 (14%) versus 5/27 (19%), respectively, received other types of chemotherapy and 5 patients did not receive any chemotherapy. The differences in recurrence rate were not statistically significant between any two subgroups in terms of adjuvant chemotherapy (Table 1).

**Table 1:** Demographic and clinical features of the study population

Variable	Non-recurrent, n (%)	Recurrent, n (%)	P value^a^
Age (years)			
≤57	28 (49)	16 (59)	0.385
>57	29 (51)	11 (41)	
Sex			
Male	36 (63)	17 (63)	0.986
Female	21 (37)	10 (37)	
Race			
White	39 (68)	16 (59)	0.409
Non-white	18 (32)	11 (41)	
Location			
Cecum/transverse/descending colon	19 (33)	2 (7)	**0.029^b^**
Ascending colon	17 (30)	10 (37)	
Sigmoid	21 (37)	15 (56)	
CEA (μg/L)			
≤5	52 (91)	22 (82)	0.279
>5	5 (9)	5 (19)	
Tumor stage			
IIA	6 (11)	3 (11)	0.191^b^
IIB, IIC	2 (4)	0 (0)	
IIIA	4 (7)	0 (0)	
IIIB	33 (58)	13 (48)	
IIIC	12 (21)	11 (41)	
Tumor differentiation			
Moderate to well, well	44 (77)	22 (82)	0.655
Moderate to poor, poor	13 (23)	5 (19)	
Lymph vascular invasion			
No	45 (79)	23 (85)	0.497
Yes	12 (21)	4 (15)	
Perineural invasion			
No	42 (74)	19 (70)	0.750
Yes	15 (26)	8 (30)	
Margin positive			
No	23 (40)	8 (30)	0.342
Yes	34 (60)	19 (70)	
Lymph nodes resected			
2-10	32 (56)	15 (56)	0.664
11-25	5 (9)	1 (4)	
25-65	20 (35)	11 (41)	
Lymph nodes involved			
0	7 (12)	3 (11)	**0.030**
1-5	40 (70)	12 (44)	
>5	10 (18)	12 (44)	
Adjuvant chemotherapy			
None or missing	2 (4)	3 (11)	0.424
FOLFOX	47 (82)	19 (70)	
Other	8 (14)	5 (19)	

a Two-sided Chi-square test.

b Fisher's exact test.

**Figure 1:**
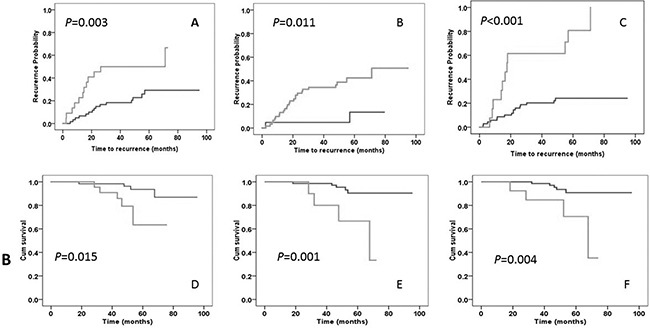
Kaplan-Meier plot of time to tumor recurrence (panels A-C) and overall survival duration (panels D-F) Panels A, B, and C represent comparisons of time to recurrence by lymph node (≤5 vs. >5 positive nodes), tumor location (cecum/transverse/left vs sigmoid/right colon), and mir-4772-3p (< vs. ≥27.88 ΔCT) status, respectively. Panels D, E, and F represent comparisons of overall survival duration by lymph node (≤5 vs. >5 positive nodes), CEA (≤5 vs. >5 mg/L), and mir-4772-3p (< vs. ≥27.88 ΔCT) status, respectively. *P* values are from a log-rank test.

The time (mean ± SD) between tumor resection and blood collection was 44.5±15.5 days for non-recurrent patients versus 45.0±14.0 days for recurrent patients (*P*=0.889). The total follow-up duration (from date of diagnosis to last follow-up) was 56.0±16.7 and 51.6±14.0 months for non-recurrent and recurrent patients, respectively (*P*=0.241). The median time from colon resection to the development of recurrent disease was 17.6 months (interquartile range: 8.7-26.3 months). Nine of the 27 recurrent patients and none of the 57 non-recurrent patients have died at the time of last follow-up; all deaths were related to colon cancer. Cox regression analyses of time to tumor recurrence and overall survival demonstrated that tumor location and CEA levels were significant clinical predictors (Figure 1).

The presence of exosome populations in the serum extracts was confirmed by scanning electron microscopy (Supplementary Figure S1) and expression of canonical exosome surface markers including CD9, CD63, and CD81 as detected by Western blots (Supplementary Figure S2). Nanoparticle-tracking analysis showed that the median and mean size of the particles was 74.5 and 81.1, respectively. A concentration of 2.1 x10^9^ to 4.8 ×10^11^ particles per ml of serum was detected in the four tested serum samples (Supplementary Figure S3).

The quality control data of the eight pooled serum exosomal RNA samples are presented in Supplementary Figure S4. MiRNAs comprised 6% to 20% of the total RNAs. RNA-seq showed that all samples had more than 20 million total reads (Supplementary Table S1). The four pooled samples from non-recurrent patients (numbers 1-4) had higher numbers (mean=584) of known miRNAs with ≥5x coverage than did those from recurrent patients (numbers 5-8) (mean=502). In the differential expression analysis, two pooled samples (numbers 2 and 6) were excluded because they had either the lowest amount of miRNA content (Supplementary Figure S4) or the smallest number of mature miRNAs detected (Supplementary Table S1). A differential expression analysis between the non-recurrent and recurrent patients was conducted using both edgeR and DeSeq software; the top 13 differentially expressed miRNAs are listed in Table 2. An edgeR analysis identified 145 differentially expressed mature miRNAs with *P*<0.05 and 50 with FDR<0.05 (Supplementary Table S2). On the other hand, an analysis using DeSeq detected only 11 miRNAs with FDR<0.10 (two with FDR<0.05) (Table 2). Table 2 lists the miRNAs with FDR<0.05 (by edgeR) and the highest fold change between recurrent and non-recurrent patients. An Ingenuity Pathway Analysis of the 145 miRNAs with nominal significance by edgeR identified several cellular functions that were over-represented by the differentially expressed miRNAs, i.e., cellular development, cellular growth and proliferation, cell cycle, cellular movement, and cell death and survival (Supplementary Table S3).

**Table 2:** Top differentially expressed miRNAs, as detected by RNA sequencing

MiRNA	EdgeR			DeSeq		
	log2ratio^a^	*P* value	FDR	log2ratio	*P* value	FDR
hsa-miR-451a	−1.41	7.19E-07	0.0004	−1.45	5.72E-05	0.0327
hsa-miR-107	−1.32	2.74E-06	0.0008	−1.36	1.26E-04	0.0361
hsa-miR-4732-5p	−1.69	5.27E-06	0.0010	−1.73	6.47E-04	0.0683
hsa-miR-3688-3p	−2.56	1.43E-05	0.0016	−2.64	8.73E-04	0.0683
hsa-miR-485-3p	1.63	1.43E-05	0.0016	1.60	4.11E-04	0.0681
hsa-miR-4306	−1.47	5.37E-05	0.0051	−1.51	8.98E-04	0.0683
hsa-miR-486-5p	−1.16	7.81E-05	0.0062	−1.20	4.77E-04	0.0681
hsa-miR-3143	−1.56	8.90E-05	0.0062	−1.62	1.88E-03	0.0975
hsa-miR-4433b-5p	1.57	9.72E-05	0.0062	1.55	9.56E-04	0.0683
hsa-miR-96-5p	−1.16	3.33E-04	0.0150	−1.20	1.34E-03	0.0784
hsa-miR-370-3p	1.29	3.42E-04	0.0150	1.25	1.37E-03	0.0784
hsa-miR-151b	−1.31	2.65E-04	0.0150			
hsa-miR-451b	−5.68	3.24E-04	0.0150			

Top down-regulated				Top up-regulated			
MiRNA	log2ratio	*P* value	FDR	MiRNA	log2ratio	*P* value	FDR
hsa-miR-451b	−5.68	3.24E-04	0.0150	hsa-miR-370-3p	1.29	3.42E-04	0.0150
hsa-miR-4482-3p	−4.54	9.41E-04	0.0256	hsa-miR-758-3p	1.35	3.09E-03	0.0414
hsa-miR-548ac	−3.72	1.11E-03	0.0287	hsa-miR-136-5p	1.40	3.45E-03	0.0437
hsa-miR-3688-3p	−2.56	1.43E-05	0.0016	hsa-miR-429	1.42	3.14E-03	0.0414
hsa-miR-3200-5p	−2.21	7.30E-04	0.0236	hsa-miR-654-5p	1.46	7.91E-04	0.0236
hsa-miR-4732-5p	−1.69	5.27E-06	0.0010	hsa-miR-369-5p	1.47	2.72E-03	0.0409
hsa-miR-3143	−1.56	8.90E-05	0.0062	hsa-miR-4433b-5p	1.57	9.72E-05	0.0062
hsa-miR-4448	−1.52	2.27E-03	0.0403	hsa-miR-485-3p	1.63	1.43E-05	0.0016
hsa-miR-3143	−1.56	8.90E-05	0.0062	hsa-miR-337-3p	1.85	1.86E-03	0.0380

a Fold difference between the recurrent and non-recurrent groups. A negative value indicates under-expression, and a positive value indicates over-expression in the recurrent group.

We tested the expression of 10 differentially expressed miRNAs (Table 3) in 84 individual samples using a qRT-PCR assay. MiR-4772-3p showed 5.91-fold lower expression in recurrent than in non-recurrent patients, with a *P* value of 0.002 by both the parametric *t* test and non-parametric Mann-Whitney U-tests (Table 3), which was statistically significant after Bonferroni correction. MiR-4732-5p was upregulated in the recurrent group by 1.8-fold, and the *P* value was 0.044 and 0.144 in the parametric and non-parametric tests, respectively. Mir-451b was under-expressed by 2.95-fold in the recurrent group, but the difference did not reach statistical significance (*P*=0.093 and 0.074 in the U test and *t* test, respectively). The expression of the other seven markers did not significantly differ between the two groups.

**Table 3:** MiRNA expression by qRT-PCR in non-recurrent and recurrent patients

MiRNA	Non-recurrent, n=57	Recurrent,^a^ n=27	Fold change^b^	*P* value^c^	*P* value^d^
**hsa-miR-4732-5p**	10.40±3.92	8.60±3.45	**1.80**	**0.144**	**0.044**
**hsa-miR-451a**	4.79±4.31	3.90±3.03	0.89	0.569	0.336
**hsa-miR-3200-3p**	22.50±7.10	22.92±10.11	−0.42	0.882	0.845
**hsa-miR-3143**	32.57±6.71	34.34±4.63	−1.78	0.524	0.217
**hsa-miR-485-3p**	12.80±4.02	11.04±7.80	1.76	0.129	0.276
**hsa-miR-4772-3p**	19.71±5.52	25.62±8.71	**-5.91**	**0.002**	**0.002**
**hsa-miR-1255b-5p**	8.09±3.38	8.42±3.68	−0.33	0.966	0.686
**hsa-miR-1180-3p**	13.02±6.14	13.12±7.49	−0.10	0.629	0.950
**hsa-miR-451b**	22.84±6.51	25.80±7.84	**-2.95**	**0.093**	**0.074**
**hsa-miR-3200-5p**	13.65±3.80	14.22±2.69	−0.58	0.248	0.480

a Relative expression presented as mean ± standard deviation of ΔCT.

b Fold difference (2^−ΔΔCT^) between the non-recurrent and recurrent groups. A negative value indicates under-expression, and a positive value indicates over-expression in the recurrent group.

c Non-parametric Mann-Whitney U-tests.

d Student's *t* test.

A logistic regression model was built using tumor recurrence status as the dependent variable. The ROC curve was constructed for miR-4772-3p as a predictor of tumor recurrence. Compared to tumor location and lymph node metastasis, the two known clinical predictors for recurrence, miR-4772-3p had better performance in both tests, i.e. a smaller *P* value for OR and a greater AUC (Table 4). Using the 60th percentile of the ΔCT value (27.88) of miR-4772-3p in the recurrent group as the cut-off, patients with a lower level of expression (higher ΔCT value) of miR-4772-3P had a significantly increased risk of tumor recurrence (OR: 11.3, 95% CI: 2.38-53.2, *P*<0.001) (Table 4). An ROC analysis was performed on miR-4774-3p to discriminate recurrent from non-recurrent patients; the AUC was 0.72 (95% CI: 0.59-0.85, *P*=0.001), and the sensitivity and specificity were 78.6% and 77.1%, respectively (Table 4).

**Table 4:** Logistic regression and ROC analyses of risk of tumor recurrence

Variable	Non-recurrent n (%)	Recurrent n (%)	Logistic regression OR (95% CI)	*P* value	ROC, AUC (95% CI)	*P* value
miR-4772-3p						
<27.88	54 (95)	16 (59)	1.0			
≥27.88	3 (5)	11 (41)	11.3 (2.38-53.2)	0.002	0.72 (0.59-0.85)	0.001
Tumor site						
Cecum/transverse/descending	19 (33)	2 (7)	1.0			
Ascending/sigmoid	38 (67)	25 (93)	6.02 (1.14-31.8)	0.034	0.63 (0.51-0.75)	0.062
Lymph node positive						
≤5	47 (83)	15 (56)	1.0			
>5	10 (17)	12 (44)	2.77 (0.84-9.17)	0.095	0.65 (0.51-0.78)	0.034

Mir-4772-3p was dichotomized using the 60th percentile of the ΔCT value of recurrence patients.

The variables mir-4772-3p expression, CEA level, and lymph node status were all included in the multivariate logistic regression model.

CEA and the AJCC stage were dropped due to insignificance.

A Kaplan-Meier plot and the log-rank test showed that patients with a lower level of miR-4772-3p expression (≥27.88 ΔCT) had a significantly shorter time to recurrence (mean ± standard error=32.5±7.4 months) than did those with a higher expression level (77.0±3.9), *P<*0.001 (Figure 1). Tumor location and CEA levels were also significantly associated with a shorter time to recurrence and increased risk of recurrence (Figure 1 and Table 5). With a low frequency of death (n=9), the median overall survival duration could not be calculated; however, the mean ± standard error for overall survival was 87.4±2.6 months, which was significantly shorter in patients with low miR-4772-3p expression (60.9±5.3) than in those with high expression (90.9±2.0), *P*=0.004 (Figure 1). A multivariate Cox regression model that included miR-4772-3p (< vs. ≥27.88 ΔCT), tumor location (cecum/transverse/descending colon vs. ascending/sigmoid), and serum CEA levels (≤ 5 vs. >5 mg/L) revealed that patients with reduced expression of miR-4772-3p had a 5.48-fold increased risk of tumor recurrence (95% CI: 2.49-12.1), *P*<0.001 (Table 5). Furthermore, low miR-4772-3p and high CEA levels, but not tumor location, were significantly related to an increased risk of death in this patient population (Table 5).

**Table 5:** Analysis of time to recurrence and overall survival duration by Cox regression analysis

Variable	# of recurrences/# of patients	Time to recurrence (mean ± standard error)^a^	Risk of recurrence, HR (95% CI)	# of deaths/# of patients	Survival duration (mean ± standard error)^a^	Risk of death, HR (95% CI)
miR-4772-3p						
<27.88	16/70	77.0±3.9	1.0	5/70	90.9±2.0	1.0
≥27.88	11/14	32.5±7.4	5.48 (2.49-12.1)	4/14	60.9±5.3	6.19 (1.50-25.5)
*P* value			<0.001			0.012
Tumor site						
Cecum/transverse/descending	2/21	73.7±4.0	1.0	1/21	79.2±2.1	1.0
Ascending/sigmoid	25/63	62.1±6.0	7.42 (1.64-33.5)	8/83	86.7±2.9	7.37 (0.74-73.3)
*P* value			<0.001			0.089
CEA (mg/L)						
≤5	22/74	70.3±4.4	1.0	5/74	90.6±2.1	1.0
>5	5/10	43.2±8.8	2.87 (1.04-7.94)	4/10	59.0±5.4	9.57 (2.29-39.9)
*P* value			0.042			0.002

a Because median time to recurrence and overall survival duration could not be calculated for the low-risk groups, means and standard error values are presented for all groups.

The distribution of tumor recurrence rate by subgroups of chemotherapy, tumor stage, and miR-4772-3p expression levels were presented in Supplementary Table S4. The number of patients with stage II disease, patients that did not receive any adjuvant therapy, or other adjuvant chemotherapy other than FOLFOX were too small to make a meaningful comparison. The only significant difference in tumor recurrence rate by miR-4772-3p expression level was observed in the group of patients with stage III tumor who had received adjuvant FOLFOX. Patients with a low level of miR-4772-3p expression had a much higher tumor recurrence rate (90%) than those with a high level of expression (18%) (*P*<0.001).

Using TargetScanHuman version 7.0 software [20], we identified several potential target genes of miR-4772-3p; the top 20 are listed in Supplementary Table S5. The top two genes were *TFRC* (transferrin receptor) and *RTN4* (reticulon 4), with total context^++^ scores of −1.2 and −1.03, respectively. Other genes included *RAB9A* (a member of the *ras* oncogene family), with a score of 0.93.

## DISCUSSION

The aims of this study were to identify specific serum exosomal miRNAs that can serve as prognostic biomarkers of recurrent stage II and stage III colon cancer as well as predictive markers for response to adjuvant therapy. In this study of 84 stage II and stage III colon cancer patients by RNA-seq and RT-PCR, the baseline level of serum exosomal miR-4772-3p was significantly lower in the 27 patients with recurrent disease than in that of the 57 patients without recurrence. The lower level of miR-4772-3p expression at baseline was significantly associated with a 5.48-fold shorter time to recurrence and 6.19-fold increased risk of death after adjusting for other clinical factors. Because this marker was readily measurable in the circulating blood at baseline, before any adjuvant therapy, it has great potential value for identifying stage II and stage III colon cancer patients who have a high risk of tumor recurrence. Although we observed supporting evidence for miR-4772-3p as a potential predictive marker in patients who received FOLFOX adjuvant therapy, because 94% of the 84 patients underwent adjuvant chemotherapy, this study could not distinguish whether miR-4772-3p was truly a predictive marker for response to FOLFOX or it is a prognostic marker for tumor recurrence only. A future study is required to clarify this issue and is being planned for validation.

Compared to microarray technology, RNA-seq has the advantage of higher sensitivity and the ability to detect new miRNAs that have not been previously reported [21]. The current study using RNA-seq identified a large number of differentially expressed mature miRNAs between recurrent and non-recurrent colon cancer patients. Because of the cost-effectiveness consideration, only a small number of the top hit miRNAs were evaluated in the validation study. A quantitative RT-PCR assay confirmed that one of the selected markers, miR-4772-3p, was significantly under-expressed in recurrence patients; this difference was statistically significant after adjusting for multiple testing. Logistic regression modeling and a ROC analysis demonstrated that this miRNA had better performance than did other clinical factors, such as tumor location and lymph node metastasis, in predicting tumor recurrence. MiR-4772-3p has previously been reported to be differentially expressed in tumor versus normal breast tissues [22] and in patients with and without Alzheimer disease [23]. Although the functional significance of miR-4772-3p remains unknown, a number of genes have been predicted to be targets of this miRNA, with high context scores and high relevance to tumor progression (Supplementary Table S5). For example, the *RTN4* (aka *NOGO*) gene has been implicated in lung cancer [24], hepatocellular carcinoma [25], and cervical cancer [26] and has diverse roles in epithelial-mesenchymal transition, cell adhesion, and migration [27]. The *RAB9A* gene is an important member of the *ras* oncogene superfamily. Other genes are involved in membrane trafficking, inflammation, apoptosis, and mitochondrial functions, which are all involved in tumor progression and metastasis.

Exosomes have the capacity to protect miRNAs [28] and other cellular contents from degradation in circulation and function as carriers to transmit the donor cells' contents to recipient cells [29]. Thus, serum exosomal miRNA profiles may reflect the condition of tumor cells, and under-expression of miR-4772-3p may lead to the overexpression of oncogenes and the genes that promote tumor progression and metastasis [30]. Further investigation into the regulatory effects of miR-4772-3p on these genes and their role in CRC progression is warranted.

The strength of our study includes the prospective collection of blood specimens from a relatively homogenous study population. Most studies of this kind used blood samples collected after tumor recurrence, which may lead to the discovery of biomarkers that are either depending on the tumor volume or affected by adjuvant treatment. Using blood samples collected after tumor resection but before receiving any adjuvant chemotherapy is clinically relevant since this is the juncture when clinical decision is made regarding the use of adjuvant chemotherapy. Because all patients were treated at MD Anderson under the same treatment regimen, and several criteria were applied at patient enrollment, we expect minimum selection bias. The limitations of the study include small number of miRNAs in the validation study and lack of miRNA expression data in the tumor tissues. It is essential that our findings be validated in other study populations with a larger sample size. Correlating serum exosomal miRNA expression with that of tumor tissues is also highly desirable.

Overall, this is the first study to report serum exosomal miRNA profiling by RNA-seq and qRT-PCR in stage II and III colon cancer patients. Our findings identified a potential prognostic/predictive marker of tumor recurrence. Further validation of these findings in tumor tissue samples and additional blood samples are underway. If confirmed in a larger patient population, the level of miR-4772-3p could be helpful in determining the role of adjuvant FOLFOX therapy as well the interval of diagnostic imaging in patients with stage II or III colon cancer who are considered to be at high risk following surgical resection. Furthermore, if validated, miR-4772-3p should be evaluated in patients undergoing metastatic surgical resection, in which the role of adjuvant chemotherapy remains a subject of debate. In addition, a further functional analysis of miR-4772-3p may provide the fundamental information required for future targeted therapy.

## MATERIALS AND METHODS

### Study population

Patients with stage II and stage III colon cancer were prospectively recruited from the Colorectal Cancer Center at The University of Texas MD Anderson Cancer Center (Houston, Texas) from September 2006 to April 2011. The study design was approved by the institutional review board of MD Anderson. All patients signed an informed consent form for interviewing and for collection of blood and residual tissue samples for research. All patients had histologically proven AJCC (version 7) stage II (T_3-4_N_0_M_0_) or III (T_x_N_1-3_M_0_) adenocarcinoma of the colon and were aged 18 years or older. Patients with a known history of familial adenomatous polyposis, hereditary non-polyposis CRC, other hereditary polyposis syndromes (e.g., Muir Torre or Gardner's syndrome); a diagnosis of rectal cancer; or prior malignancies (excluding non-melanoma skin neoplasms) over the past 5 years were excluded. Patients agreed to be followed up for a surveillance period of up to 2 years. Peripheral blood samples were collected within 12 weeks of surgical resection and before the initiation of adjuvant chemotherapy. The primary endpoint was the occurrence of local recurrent or distant metastasis, determined radiologically or histologically.

### Exosome isolation and characterization

After blood was drawn, serum was immediately separated from the cellular fraction by centrifugation at 3000g for 10 min at ambient temperature and stored at −80°C until use.

Exosomes were extracted from 250-μl serum samples using the ExoQuick kit (SBI, Mountain View, CA, USA) according to the manufacturer's instructions. An aliquot of the exosomes was fixed in Formaldehyde/Glutaraldehyde, 2.5% each in 0.1M Sodium Cacodylate Buffer, pH 7.4 for 15 minutes for electronic microscopy imaging. Digital images were obtained using the AMT Imaging System (Advanced Microscopy Techniques Corp., Danvers, MA) by the High Resolution Electron Microscopy Facility at MD Anderson. The remaining exosome pellets were suspended in PBS and serially diluted to the optimum dynamic range of the Zetaview nanoparticle analyzer (Particle Metrix, Diessen, Germany) for measurement of size and particle density. A separate exosome preparation from 250-μl serum samples was used to examine exosome markers by Western blotting with the following primary antibodies 1:1000 CD63, 1:1000 CD81, and 1:1000 CD9 (CD63, CD81, and CD9 rabbit anti-human from EXO-AB System bioscience). The detailed experimental protocols of these assays have previously been described [31].

### RNA extraction and RNA-seq

Exosomal RNA was extracted using the SeraMir exosome RNA kit (SBI, Mountain View, CA, USA) according to the manufacturer's instructions. The quality and quantity of the RNA was determined using the Small RNA Chip and Agilent Bioanalyzer 2100 (Agilent Technologies, Santa Clara, CA). Samples with an RNA integrity number of >7.0 were eligible for RNA-seq. Twenty patients each in the recurrent and non-recurrent groups were matched by age, sex, race, and tumor stage. Five RNA samples from each group were pooled, and 8 were subjected to RNA-seq. The library was prepared using the NEBNext Small RNA Library Prep Set for Illumina, following the manufacturer's protocol (New England BioLabs, Ipswich, MA). The constructed libraries were quality checked using a bioanalyzer and pooled, followed by size selection in a range of 130 bp and 166 bp using a 3% agarose gel cassette on a BluePippin (Saga Science, Beverly, MA). RNA-seq was conducted on an Illumina HiSeq 2000 at the Science Park NGS facility. The quality of the sequencing data was analyzed by the bioinformatics team associated with the sequencing core. In brief, the reads were first trimmed by removing the adaptor sequences and then mapped to human miRNAs from miRBase Release 21 [32] using miRDeep2 (version 2.007) [33], which used the short read aligner Bowtie (version 1.0.0) [34] internally. The number of reads in each miRNA was enumerated by miRDeep2, and differences in expression between groups were statistically assessed by DeSeq (version 1.16.0) [35] and edgeR (version 3.6.1) [36]. A pathway analysis was conducted on the differentially expressed miRNAs with nominal significance using Ingenuity Pathway Analysis software (Ingenuity Systems, Redwood City, CA).

### Validation test by quantitative real-time PCR

Ten differentially expressed miRNA species were selected for validation on the basis of the criteria of >1.5-fold change, *P*<0.05, and false-positive reporting probability <0.05 or on the results of a literature search suggesting relevance to human cancer. An RT-PCR validation assay was conducted in samples from all 84 patients using an ABI PRISM 7900 HT real-time RT-PCR system (Applied Biosystems, Foster City, CA). In brief, 5 ml of exosomal RNA was reverse transcribed to cDNA using small RNA assays and miRNA-specific primers (Thermo Fisher Scientific, Waltham, MA), according to the manufacturer's instructions. The primer sequences for each tested miRNA are presented in Supplementary Table S6. MiR-16 was included as an internal standard, as suggested by the results of previous studies [37]. Each sample was analyzed in triplicate in an RT-PCR assay, and the mean threshold cycle (Ct) value of the triplicates was used in the final data analysis. The RT-PCR results were first normalized to the Ct value of miR-16, referred to as ΔCt. The fold change in miRNA expression in the recurrent group compared to that in the non-recurrent group was expressed as 2^−Δ ΔCt^. The potential target genes of miRNAs were predicted using TargetScan 7.0 [20].

### Statistical analysis

Student's *t*-test and nonparametric Mann-Whitney U-tests were used to compare the differences in serum miRNA expression between the recurrent and non-recurrent groups according to the variable distribution. The predictive value of the markers was evaluated by constructing logistic regression models, Cox proportional hazard regression models, and receiver operating curves (ROCs). Time to recurrence and overall survival curves were generated using the Kaplan-Meier method, and the statistical significance of the differences was determined using Gehan's modification of the Wilcoxon signed-rank test. The risk of recurrence and risk of death were estimated using univariate and multivariate hazard ratios (HRs), and 95% confidence intervals (CIs) were calculated using Cox proportional hazard models.

All tests used SPSS software 21.0 (SPSS, Inc., Chicago, IL, USA), and a P<0.05 was considered statistically significant. To control the false-positive detection rate associated with multiple testing, a Bonferroni correction was applied. A *P*<0.005 (0.05/10 [number of samples tested]) was considered as multiple comparison adjusted significant.

## SUPPLEMENTARY FIGURE AND TABLES




