# Supplementary material for: Serum exosomal miR-4772-3p is a predictor of tumor recurrence in stage II and III colon cancer
Source: Oncotarget. 2016 Oct 24;7(46):76250–60. doi: 10.18632/oncotarget.12841 (PMC5342811; doi:10.18632/oncotarget.12841)
Supplement: Supplementary file 1 [file oncotarget-07-76250-s001.pdf]

Serum exosomal miR-4772-3p is a predictor of tumor recurrence in stage II and III colon cancer

SUPPLEMENTARY FIGURE AND TABLES

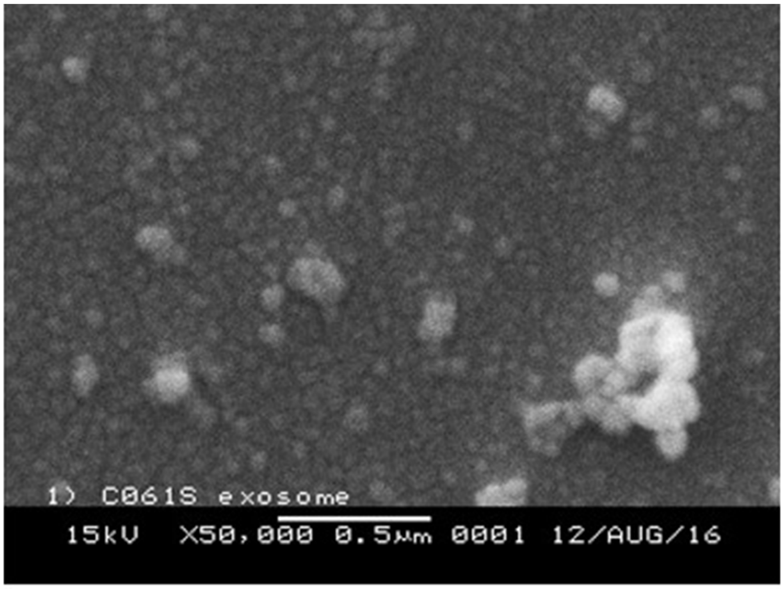

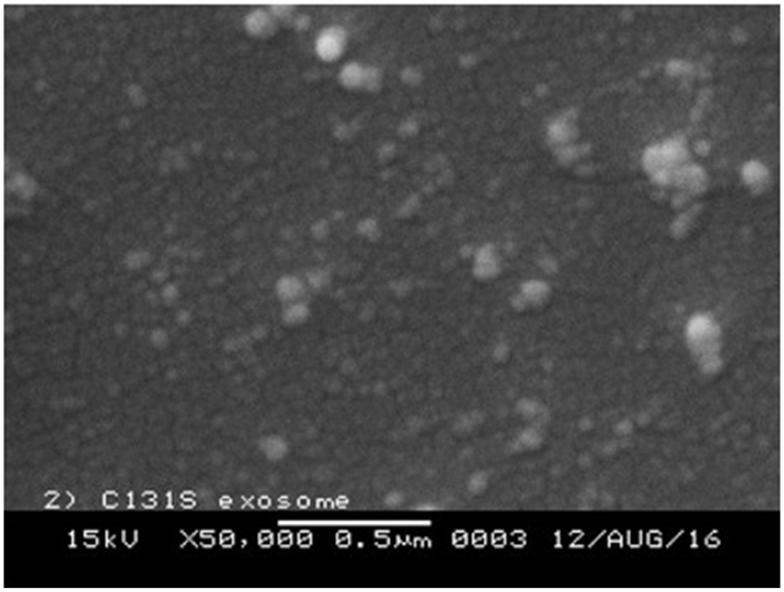

Supplementary Figure S1: Scanning electronic microscopy images of exosomes isolated from serum samples of colon cancer patients.

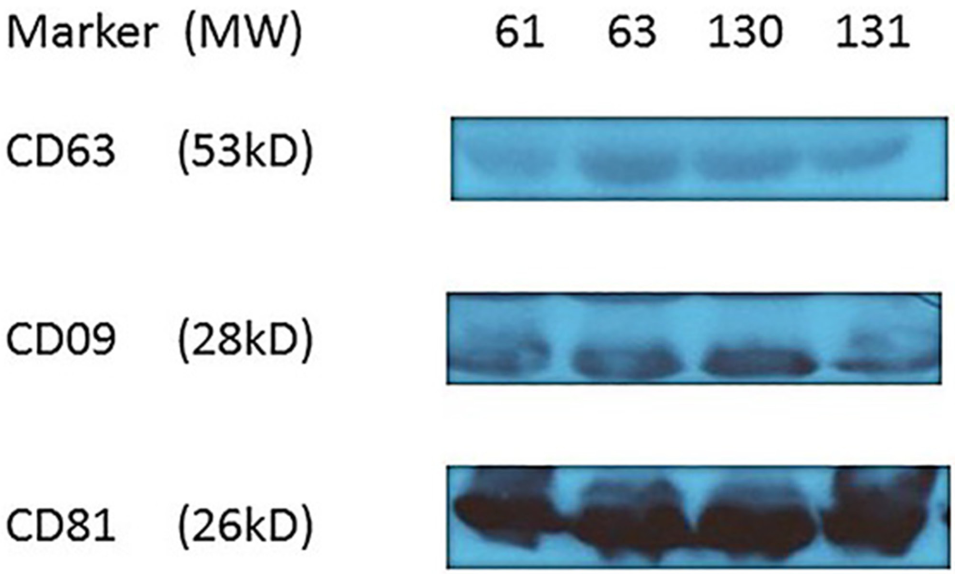

Supplementary Figure S2: Expression of exosome surface markers CD9, CD61 and CD83 in serum exosome extracts from 4 colon cancer patients.

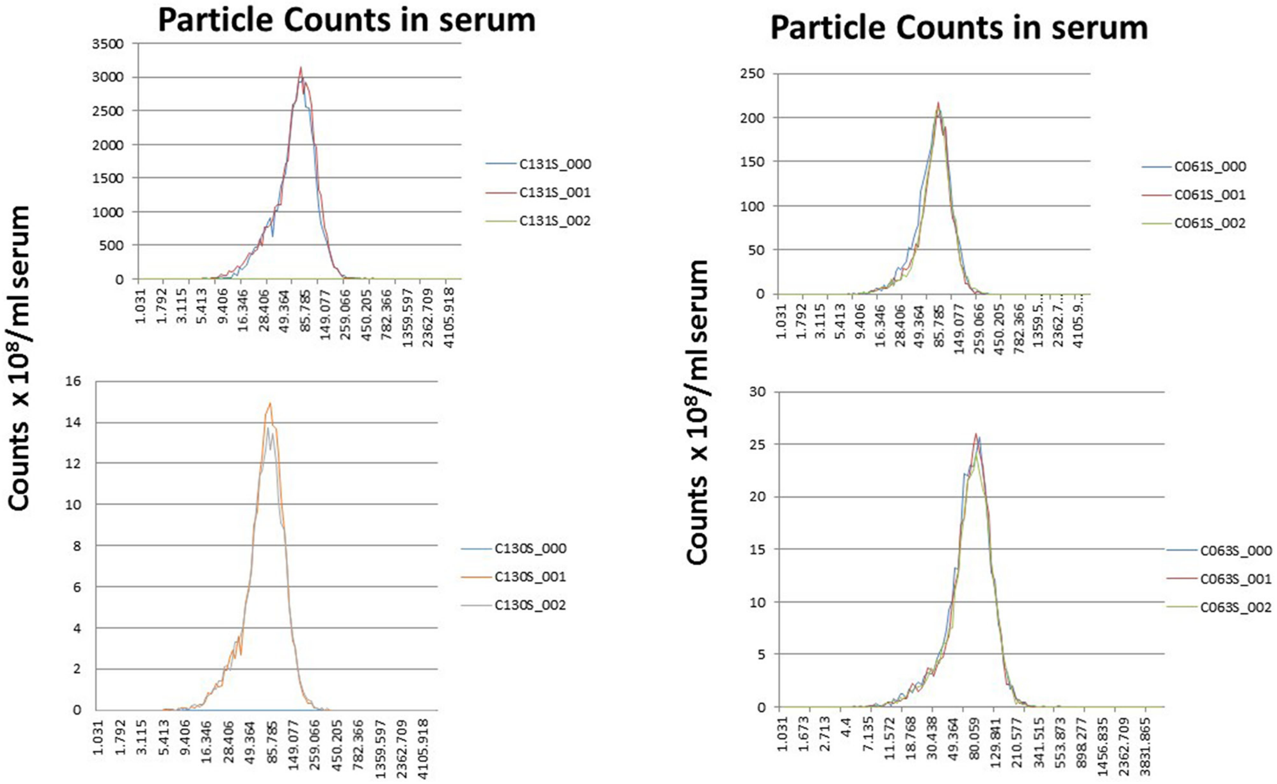

Supplementary Figure S3: Particle counts and size distribution in serum exosome preparation of 4 colon cancer patients as determined by nanoparticle tracking analysis.

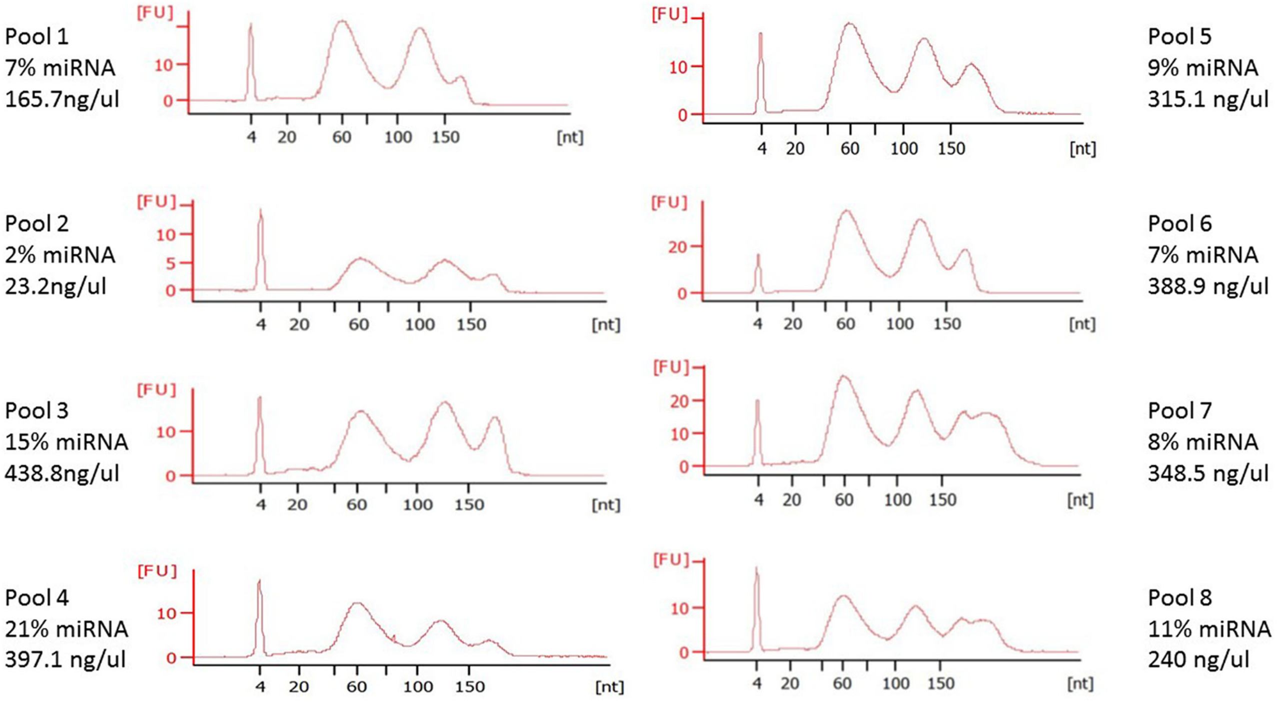

Supplementary Figure S4: Exosomal total RNA quality and percentage of microRNAs in each pooled sample.

Supplementary Table S1: Information on miRNA-sequencing data

Sample	Total Reads	Trimmed Reads	Too Short After Trimming (<17bps)	Reads sent to Aligner	Aligned Reads	Precursor miRNA Reads	Mature miRNA Reads	Known miRNA with >= 5x coverage
1	29766145	24346044	4424571	25341574	10626057	2828	3200648	605
2	23115699	19610038	4170224	18945475	7771578	1941	2368380	578
3	24612665	20444473	2762484	21850181	9374227	2286	3345935	552
4	21345251	18194261	2702998	18642253	9383931	3290	3758687	600
5	22433569	18508709	4009229	18424340	5034894	1153	1178083	497
6	21604833	17723515	3809950	17794883	6610641	1499	1366346	482
7	23020710	19009264	3124714	19895996	9637725	2573	1713088	521
8	20421669	17082159	4011159	16410510	5811217	1880	1417724	511

Sample 1-4: non-recurrent; sample 5-8: recurrent

Supplementary Table S2: Differentially expressed miRNAs with nominal significance.

See Supplementary File 1

Supplementary Table S3: Results of IPA analysis on 146 differentially expressed miRNAs

**Molecular and cellular functions**		
**Name**	**P-values**	**#/molecules**
Cellular Development	4.78E-02 - 4.60E-06	27
Cellular Growth and Proliferation	4.78E-02 - 4.60E-06	25
Cell Cycle	4.87E-02 - 8.44E-06	7
Cellular Movement	2.82E-02 - 2.38E-05	14
Cell Death and Survival	4.71E-02 - 6.55E-04	19
**Top Analysis Ready Molecules**		
**Molecules**	**P Value**	
miR-451a (and other miRNAs w/seed AACCGUU)	7.19E-07	
miR-103-3p (and other miRNAs w/seed GCAGCAU)	2.74E-06	
miR-4732-5p (miRNAs w/seed GUAGAGC)	5.27E-06	
miR-3688-3p (and other miRNAs w/seed AUGGAAA)	1.43E-05	
miR-485-3p (and other miRNAs w/seed UCAUACA)	1.43E-05	
miR-185-5p (and other miRNAs w/seed GGAGAGA)	5.37E-05	
miR-486-5p (and other miRNAs w/seed CCUGUAC)	7.81E-05	
miR-3143 (miRNAs w/seed UAACAUU)	8.90E-05	
miR-4433b-5p (miRNAs w/seed UGUCCCA)	9.72E-05	
miR-151-5p (and other miRNAs w/seed CGAGGAG)	2.65E-04	

Supplementary Table S4: Association of MIR-4772-3P and tumor recurrence by tumor stage and chemotherapy

Chemotherapy[a]	Tumor Stage	MIR-4772-3P Expression[b]	Non-recurrent n (%)	Recurrent n (%)	*P* value
**None**	Stage II	High	0	1 (100)	
		Low	0	0	NA[c]
	Stage III	High	1 (33)	2 (67)	
		Low	1 (100)	0	1.0[d]
	Combined	High	1 (25)	3 (75)	
		Low	1 (100)	0	0.17
**FOLFOX**	Stage II	High	12 (86)	2 (14)	
		Low	0	1 (100)	0.20[d]
	Stage III	High	34 (82)	8 (18)	
		Low	1 (10)	8 (90)	<0.001
	Combined	High	46 (82)	10 (18)	
		Low	1 (10)	9 (90)	<0.001
**Other types**	Stage II	High	2 (100)	0	
		Low	1 (50)	1 (50)	1.0[d]
	Stage III	High	5 (63)	3 (37)	
		Low	0 (56)	1 (44)	0.44[d]
	Combined	High	7 (70)	3 (30)	
		Low	1 (33)	2 (67)	0.25

[a]None: no chemotherapy; FOLFOX: Oxaliplatin with fluorouracil (5FU) and folinic acid; Other types: XELOX (capecitabine plus oxaliplatin), capecitabine or 5FU alone, and FOLFIRI (Irinotecan with 5FU and folinic acid.
[b]High: MIR-4772-3P ΔCT value <27.88; Low: : MIR-4772-3P ΔCT value ≥ 27.88.
[c]NA: P value could not be calculated because two or more cells are empty.
[d]P values were calculated using Fisher's Exact test (two sided).

Supplementary Table S5: Potential gene targets of miR-4772-3p

Gene	Gene name	Total context++ score
TFRC	transferrin receptor	-1.2
RTN4	reticulon 4	-1.03
CARD16	caspase recruitment domain family, member 16	-0.71
IL13	interleukin 13	-0.7
TTC9C	tetratricopeptide repeat domain 9C	-0.75
RAC1	ras-related C3 botulinum toxin substrate 1 (rho family, small GTP binding protein Rac1)	-0.68
MRPL19	mitochondrial ribosomal protein L19	-0.83
PIGY	phosphatidylinositol glycan anchor biosynthesis, class Y	-0.65
CPEB1	cytoplasmic polyadenylation element binding protein 1	-0.64
MDH2	malate dehydrogenase 2, NAD (mitochondrial)	-0.63
COX7C	cytochrome c oxidase subunit VIIc	-0.62
SLC3A2	solute carrier family 3 (amino acid transporter heavy chain), member 2	-0.62
RAB9A	RAB9A, member RAS oncogene family	-0.93
IL36B	interleukin 36, beta	-0.6
STK31	serine/threonine kinase 31	-0.58
ZNF785	zinc finger protein 785	-0.58
RAE1	ribonucleic acid export 1	-0.56
CEP57L1	centrosomal protein 57kDa-like 1	-0.61
PSMA1	proteasome (prosome, macropain) subunit, alpha type, 1	-0.57
ITGA8	integrin, alpha 8	-0.56

The context++ score is the sum of the contribution of 14 features (See reference 20).

Supplementary Table S6: Primer sequences for the 10 miRNAs tested by RT-PCR

ID	Prime sequence (stem-loop)
**Hsa-mir-4732**	GAGGGAGCTGTAGAGCAGGGAGCAGGAAGCTGTGTGTGTCCAG CCCTGACCTGTCCTGTTCTGCCCCCAGCCCCTC
**Hsa-mir-451a**	CTTGGGAATGGCAAGGAAACCGTTACCATTACTGAGTTTAGTAA TGGTAATGGTTCTCTTGCTATACCCAGA
**Hsa-mir-3200-3p**	GGTGGTCGAGGGAATCTGAGAAGGCGCACAAGGTTTGTGTCCAA TACAGTCCACACCTTGCGCTACTCAGGTCTGCTCGTGCCT
**Hsa-mir-3143**	TAGATAACATTGTAAAGCGCTTCTTTCGCGGTTGGGCTGGAGCAA CTCTTTACAATGTTTCTA
**Hsa-mir-485-3p**	ACTTGGAGAGAGGCTGGCCGTGATGAATTCGATTCATCAAAGCGA GTCATACACGGCTCTCCTCTCTTTTAGT
**Hsa-mir-4772-3p**	GTGATTGCCTCTGATCAGGCAAAATTGCAGACTGTCTTCCCAAATA GCCTGCAACTTTGCCTGATCAGAGGCAGTCAC
**Hsa-mir-1255b-5p**	TACGGATGAGCAAAGAAAGTGGTTTCTTAAAATGGAATCTACTCT TTGTGAAGATGCTGTGAA
**Hsa-mir-1180**	GCTGCTGGACCCACCCGGCCGGGAATAGTGCTCCTGGTTGTTTCC GGCTCGCGTGGGTGTGTCGGCGGC
**Hsa-mir-451b**	TGGGTATAGCAAGAGAACCATTACCATTACTAAACTCAGTAATGGTA ACGGTTTCCTTGCCATTCCCA
**Hsa-mir-3200-5p**	GGTGGTCGAGGGAATCTGAGAAGGCGCACAAGGTTTGTGTCCAATA CAGTCCACACCTTGCGCTACTCAGGTCTGCTCGTGCCCT
