# Supplementary material for: Serum exosomal miR-4772-3p is a predictor of tumor recurrence in stage II and III colon cancer
Source: Oncotarget. 2016 Oct 24;7(46):76250–60. doi: 10.18632/oncotarget.12841 (PMC5342811; doi:10.18632/oncotarget.12841)
Supplement: Supplementary file 2 [file oncotarget-07-76250-s002.docx]

| STable 2. Differentially expressed miRNAs with nominal significance | | | | | |
| --- | --- | --- | --- | --- | --- |
| Mature.ID | logFC* | P Value | FDR |  |  |
| hsa-miR-451a | -1.41 | 7.19E-07 | 0.0004 |  |  |
| hsa-miR-107 | -1.32 | 2.74E-06 | 0.0008 |  |  |
| hsa-miR-4732-5p | -1.69 | 5.27E-06 | 0.0010 |  |  |
| hsa-miR-3688-3p | -2.56 | 1.43E-05 | 0.0016 |  |  |
| hsa-miR-485-3p | 1.63 | 1.43E-05 | 0.0016 |  |  |
| hsa-miR-4306 | -1.47 | 5.37E-05 | 0.0051 |  |  |
| hsa-miR-486-5p | -1.16 | 7.81E-05 | 0.0062 |  |  |
| hsa-miR-3143 | -1.56 | 8.90E-05 | 0.0062 |  |  |
| hsa-miR-4433b-5p | 1.57 | 9.72E-05 | 0.0062 |  |  |
| hsa-miR-151b | -1.31 | 2.65E-04 | 0.0150 |  |  |
| hsa-miR-451b | -5.68 | 3.24E-04 | 0.0150 |  |  |
| hsa-miR-96-5p | -1.16 | 3.33E-04 | 0.0150 |  |  |
| hsa-miR-370-3p | 1.29 | 3.42E-04 | 0.0150 |  |  |
| hsa-miR-660-5p | -1.00 | 4.09E-04 | 0.0167 |  |  |
| hsa-miR-144-3p | -1.09 | 4.92E-04 | 0.0187 |  |  |
| hsa-miR-1180-3p | -1.15 | 5.78E-04 | 0.0206 |  |  |
| hsa-miR-3200-5p | -2.21 | 7.30E-04 | 0.0236 |  |  |
| hsa-miR-15a-5p | -0.94 | 7.46E-04 | 0.0236 |  |  |
| hsa-miR-654-5p | 1.46 | 7.91E-04 | 0.0236 |  |  |
| hsa-miR-379-5p | 1.20 | 8.28E-04 | 0.0236 |  |  |
| hsa-miR-4482-3p | -4.54 | 9.41E-04 | 0.0256 |  |  |
| hsa-miR-548ac | -3.72 | 1.11E-03 | 0.0287 |  |  |
| hsa-miR-134-5p | 1.08 | 1.52E-03 | 0.0378 |  |  |
| hsa-miR-363-3p | -0.97 | 1.75E-03 | 0.0380 |  |  |
| hsa-miR-500a-3p | -0.97 | 1.75E-03 | 0.0380 |  |  |
| hsa-miR-320b | -0.90 | 1.84E-03 | 0.0380 |  |  |
| hsa-miR-337-3p | 1.85 | 1.86E-03 | 0.0380 |  |  |
| hsa-miR-130b-5p | 1.15 | 1.86E-03 | 0.0380 |  |  |
| hsa-miR-200c-3p | 1.05 | 2.18E-03 | 0.0403 |  |  |
| hsa-miR-411-5p | 1.28 | 2.20E-03 | 0.0403 |  |  |
| hsa-miR-423-5p | -0.92 | 2.22E-03 | 0.0403 |  |  |
| hsa-miR-4448 | -1.52 | 2.27E-03 | 0.0403 |  |  |
| hsa-miR-223-3p | 0.90 | 2.35E-03 | 0.0403 |  |  |
| hsa-miR-1255b-5p | -1.22 | 2.40E-03 | 0.0403 |  |  |
| hsa-miR-6852-5p | 1.22 | 2.55E-03 | 0.0409 |  |  |
| hsa-miR-369-5p | 1.47 | 2.72E-03 | 0.0409 |  |  |
| hsa-miR-18b-5p | -1.21 | 2.72E-03 | 0.0409 |  |  |
| hsa-miR-183-5p | -1.06 | 2.76E-03 | 0.0409 |  |  |
| hsa-miR-25-3p | -0.99 | 2.84E-03 | 0.0409 |  |  |
| hsa-miR-501-3p | -0.98 | 2.91E-03 | 0.0409 |  |  |
| hsa-miR-16-2-3p | -0.94 | 2.94E-03 | 0.0409 |  |  |
| hsa-miR-758-3p | 1.35 | 3.09E-03 | 0.0414 |  |  |
| hsa-miR-429 | 1.42 | 3.14E-03 | 0.0414 |  |  |
| hsa-miR-335-3p | 1.19 | 3.19E-03 | 0.0414 |  |  |
| hsa-miR-136-5p | 1.40 | 3.45E-03 | 0.0437 |  |  |
| hsa-miR-15b-5p | -0.82 | 3.58E-03 | 0.0445 |  |  |
| hsa-miR-744-5p | 0.89 | 3.76E-03 | 0.0448 |  |  |
| hsa-miR-654-3p | 1.04 | 3.77E-03 | 0.0448 |  |  |
| hsa-miR-1185-1-3p | 1.05 | 4.21E-03 | 0.0483 |  |  |
| hsa-miR-330-3p | 0.96 | 4.23E-03 | 0.0483 |  |  |
| hsa-miR-106b-5p | -1.02 | 4.59E-03 | 0.0500 |  |  |
| hsa-miR-493-5p | 1.06 | 4.63E-03 | 0.0500 |  |  |
| hsa-miR-3913-5p | -1.64 | 4.64E-03 | 0.0500 |  |  |
| hsa-miR-3200-3p | -2.68 | 5.00E-03 | 0.0529 |  |  |
| hsa-miR-101-3p | -0.88 | 5.13E-03 | 0.0533 |  |  |
| hsa-miR-5698 | -5.17 | 5.53E-03 | 0.0558 |  |  |
| hsa-miR-574-3p | 0.97 | 5.64E-03 | 0.0558 |  |  |
| hsa-miR-197-3p | 0.96 | 5.87E-03 | 0.0558 |  |  |
| hsa-miR-4326 | 1.60 | 5.87E-03 | 0.0558 |  |  |
| hsa-miR-92a-3p | -0.79 | 6.02E-03 | 0.0558 |  |  |
| hsa-miR-411-3p | 1.49 | 6.03E-03 | 0.0558 |  |  |
| hsa-miR-28-5p | 0.90 | 6.05E-03 | 0.0558 |  |  |
| hsa-miR-6511b-5p | -2.89 | 6.46E-03 | 0.0586 |  |  |
| hsa-miR-340-3p | 0.98 | 6.76E-03 | 0.0604 |  |  |
| hsa-miR-199a-5p | 0.93 | 6.91E-03 | 0.0607 |  |  |
| hsa-miR-16-5p | -0.86 | 7.75E-03 | 0.0670 |  |  |
| hsa-miR-493-3p | 1.04 | 8.14E-03 | 0.0694 |  |  |
| hsa-miR-382-5p | 0.96 | 8.75E-03 | 0.0735 |  |  |
| hsa-miR-1270 | -2.06 | 8.88E-03 | 0.0735 |  |  |
| hsa-miR-629-5p | -0.73 | 1.00E-02 | 0.0817 |  |  |
| hsa-miR-500b-5p | -1.82 | 1.05E-02 | 0.0831 |  |  |
| hsa-miR-331-5p | -0.96 | 1.06E-02 | 0.0831 |  |  |
| hsa-miR-320a | -0.74 | 1.07E-02 | 0.0831 |  |  |
| hsa-miR-4433b-3p | 1.15 | 1.08E-02 | 0.0831 |  |  |
| hsa-miR-7706 | -1.06 | 1.09E-02 | 0.0831 |  |  |
| hsa-miR-106b-3p | -0.92 | 1.11E-02 | 0.0833 |  |  |
| hsa-miR-378i | -0.82 | 1.16E-02 | 0.0857 |  |  |
| hsa-miR-20b-5p | -0.99 | 1.20E-02 | 0.0875 |  |  |
| hsa-miR-139-3p | 1.02 | 1.23E-02 | 0.0876 |  |  |
| hsa-miR-182-5p | -0.80 | 1.23E-02 | 0.0876 |  |  |
| hsa-miR-502-3p | -0.79 | 1.28E-02 | 0.0898 |  |  |
| hsa-miR-320c | -0.76 | 1.29E-02 | 0.0898 |  |  |
| hsa-miR-122-5p | 0.76 | 1.31E-02 | 0.0898 |  |  |
| hsa-miR-181c-3p | 1.13 | 1.32E-02 | 0.0898 |  |  |
| hsa-miR-154-5p | 1.47 | 1.37E-02 | 0.0923 |  |  |
| hsa-miR-1285-3p | -0.95 | 1.46E-02 | 0.0960 |  |  |
| hsa-miR-532-3p | -1.35 | 1.46E-02 | 0.0960 |  |  |
| hsa-miR-378c | -0.79 | 1.51E-02 | 0.0981 |  |  |
| hsa-miR-9-5p | 1.08 | 1.57E-02 | 0.0998 |  |  |
| hsa-miR-331-3p | 1.10 | 1.58E-02 | 0.0998 |  |  |
| hsa-miR-224-5p | 1.03 | 1.59E-02 | 0.0998 |  |  |
| hsa-miR-543 | 1.01 | 1.63E-02 | 0.1006 |  |  |
| hsa-miR-204-5p | 1.38 | 1.64E-02 | 0.1006 |  |  |
| hsa-miR-6773-5p | 1.21 | 1.67E-02 | 0.1016 |  |  |
| hsa-miR-643 | -2.15 | 1.71E-02 | 0.1027 |  |  |
| hsa-miR-142-3p | 0.80 | 1.82E-02 | 0.1081 |  |  |
| hsa-miR-328-3p | 0.81 | 1.88E-02 | 0.1094 |  |  |
| hsa-let-7b-5p | -0.86 | 1.90E-02 | 0.1094 |  |  |
| hsa-miR-766-3p | 1.06 | 1.91E-02 | 0.1094 |  |  |
| hsa-miR-3614-5p | 1.81 | 1.92E-02 | 0.1094 |  |  |
| hsa-miR-30e-3p | 0.67 | 1.94E-02 | 0.1099 |  |  |
| hsa-let-7g-5p | -0.74 | 1.98E-02 | 0.1108 |  |  |
| hsa-let-7c-5p | -0.70 | 2.01E-02 | 0.1115 |  |  |
| hsa-miR-485-5p | 0.97 | 2.13E-02 | 0.1171 |  |  |
| hsa-let-7i-5p | -0.65 | 2.16E-02 | 0.1175 |  |  |
| hsa-miR-193a-5p | 0.74 | 2.24E-02 | 0.1207 |  |  |
| hsa-miR-375 | 0.98 | 2.29E-02 | 0.1220 |  |  |
| hsa-miR-342-3p | -0.89 | 2.34E-02 | 0.1225 |  |  |
| hsa-miR-4792 | -0.91 | 2.34E-02 | 0.1225 |  |  |
| hsa-miR-4488 | 1.31 | 2.48E-02 | 0.1280 |  |  |
| hsa-miR-1260a | 0.98 | 2.49E-02 | 0.1280 |  |  |
| hsa-miR-486-3p | -0.84 | 2.52E-02 | 0.1280 |  |  |
| hsa-miR-4467 | -3.09 | 2.53E-02 | 0.1280 |  |  |
| hsa-miR-582-3p | 0.78 | 2.60E-02 | 0.1300 |  |  |
| hsa-miR-15b-3p | -0.67 | 2.64E-02 | 0.1313 |  |  |
| hsa-miR-29c-3p | -0.65 | 2.83E-02 | 0.1391 |  |  |
| hsa-miR-1-3p | 1.04 | 2.85E-02 | 0.1393 |  |  |
| hsa-miR-548q | -1.23 | 2.97E-02 | 0.1436 |  |  |
| hsa-miR-652-3p | -0.67 | 2.99E-02 | 0.1436 |  |  |
| hsa-miR-99b-3p | 0.90 | 3.08E-02 | 0.1465 |  |  |
| hsa-miR-548ad-5p | -0.78 | 3.18E-02 | 0.1497 |  |  |
| hsa-miR-93-5p | -0.69 | 3.20E-02 | 0.1497 |  |  |
| hsa-miR-185-5p | -0.77 | 3.24E-02 | 0.1505 |  |  |
| hsa-miR-106a-5p | -0.79 | 3.39E-02 | 0.1560 |  |  |
| hsa-miR-24-3p | 0.60 | 3.45E-02 | 0.1577 |  |  |
| hsa-miR-144-5p | -0.94 | 3.52E-02 | 0.1589 |  |  |
| hsa-miR-27b-5p | 0.99 | 3.53E-02 | 0.1589 |  |  |
| hsa-miR-24-2-5p | 0.70 | 3.58E-02 | 0.1598 |  |  |
| hsa-miR-548ak | -1.99 | 3.65E-02 | 0.1607 |  |  |
| hsa-miR-140-5p | 0.66 | 3.66E-02 | 0.1607 |  |  |
| hsa-miR-223-5p | 0.61 | 3.73E-02 | 0.1624 |  |  |
| hsa-miR-1294 | -0.84 | 3.90E-02 | 0.1685 |  |  |
| hsa-miR-4508 | -1.03 | 3.93E-02 | 0.1689 |  |  |
| hsa-miR-6516-5p | 1.41 | 4.08E-02 | 0.1725 |  |  |
| hsa-miR-125a-5p | 0.73 | 4.08E-02 | 0.1725 |  |  |
| hsa-miR-186-5p | -0.65 | 4.28E-02 | 0.1797 |  |  |
| hsa-miR-127-3p | 0.71 | 4.32E-02 | 0.1801 |  |  |
| hsa-miR-20a-5p | -0.61 | 4.35E-02 | 0.1801 |  |  |
| hsa-miR-5100 | 1.24 | 4.48E-02 | 0.1841 |  |  |
| hsa-miR-628-5p | 0.85 | 4.69E-02 | 0.1911 |  |  |
| hsa-miR-505-3p | 0.69 | 4.74E-02 | 0.1918 |  |  |
| hsa-miR-126-3p | 0.61 | 4.84E-02 | 0.1938 |  |  |
| hsa-miR-33a-5p | 0.93 | 4.85E-02 | 0.1938 |  |  |
| hsa-miR-381-3p | 0.83 | 4.94E-02 | 0.1948 |  |  |
| hsa-miR-339-5p | 0.77 | 4.95E-02 | 0.1948 |  |  |
| *Log value of fold change between recurrent versus non-recurrent group. | | | | | |
